# Metabolic Discrimination of Select List Agents by Monitoring Cellular Responses in a Multianalyte Microphysiometer

**DOI:** 10.3390/s90302117

**Published:** 2009-03-23

**Authors:** Sven E. Eklund, Roy G. Thompson, Rachel M. Snider, Clare K. Carney, David W. Wright, John Wikswo, David E. Cliffel

**Affiliations:** 1 Department of Chemistry, Vanderbilt University, VU Station B. Nashville, TN 37235, USA; E-Mails: seklund@latech.edu; r.snider@vanderbilt.edu; 2 Vanderbilt Institute for Integrative Biosystems Research and Education, Vanderbilt University, VU Station B. Nashville, TN 37235. USA; E-Mail: john.p.wikswo@vanderbilt.edu; 3 Edgewood Chemical and Biological Center, Aberdeen Proving Ground, MD 21010. USA; E-Mail: roy.thompson@us.army.mil

**Keywords:** Toxin, biotoxin, biosensors, microphysiometry

## Abstract

Harnessing the potential of cells as complex biosensors promises the potential to create sensitive and selective detectors for discrimination of biodefense agents. Here we present toxin detection and suggest discrimination using cells in a multianalyte microphysiometer (MMP) that is capable of simultaneously measuring flux changes in four extracellular analytes (acidification rate, glucose uptake, oxygen uptake, and lactate production) in real-time. Differential short-term cellular responses were observed between botulinum neurotoxin A and ricin toxin with neuroblastoma cells, alamethicin and anthrax protective antigen with RAW macrophages, and cholera toxin, muscarine, 2,4-dinitro-phenol, and NaF with CHO cells. These results and the post exposure dynamics and metabolic recovery observed in each case suggest the usefulness of cell-based detectors to discriminate between specific analytes and classes of compounds in a complex matrix, and furthermore to make metabolic inferences on the cellular effects of the agents. This may be particularly valuable for classifying unknown toxins.

## 1. Introduction

Temporal and spatial interactions between physiological pathways in a cell contain high information content regarding normal or abnormal function. Unlike biochemical assays that capture a fairly static interaction between two molecules, cell-based assays offer the ability to scan for cumulative cellular reactions that can span a wide spectrum of different physiological systems. The development and validation of cell-based assays have thus become a significant focus for high-throughput screening of lead drug compounds [1,2] and absorption, distribution, metabolism, excretion, and toxicology (ADMET) assays for preclinical drug screening [3,4].

One of the earliest efforts to automate a cell-based assay platform was the Cytosensor microphysiometer [5–7], which used a light-addressable potentiometric sensor (LAPS) to measure the extracellular acidification rate (ECAR) in cultured cells due to the build-up of acidic by-products. The Cytosensor has been used extensively to study receptor pharmacology [8,9], enzyme modulators [10], cytotoxins [11], immune effectors [12], ionophores [13], and metabolic effectors [14,15]. While often assumed to directly reflect changes in cell metabolism, an increase or decrease in ECAR alone is not a robust measure of cell metabolism: it fails to differentiate between aerobic *versus* anaerobic respiration, and a decrease in acidification rate does not necessarily indicate a decrease in cell metabolism. While Cytosensors are no longer commercially supported, a number of instruments exist in metabolic profiling laboratories and on the secondary market, and we are working with potential companies to explore a commercial multianalyte microphysiometer.

Accurate inferences of metabolic processes require measurement of multiple parameters, each possessing different analyte specificity. Recently we reported the incorporation of oxygen [16], glucose, and lactate [17] electrodes into the Cytosensor platform, making a multianalyte microphysiometer (MMP) that permits concurrent measurement of all four extracellular parameters. Changes in glucose and lactate concentrations were measured at enzyme coated platinum electrodes by the amperometric detection of hydrogen peroxide, which is a by-product of oxidase enzyme catalysis. Oxygen was measured directly by reduction at a Nafion coated platinum electrode. The rate of consumption or production of each analyte was represented by peaks during the stop-flow pump cycle as the current decreased/increased due to consumption/production of analyte. Changes in peak height or area under the peak could then be converted to changes in concentration by comparison to *in situ* calibration curves [17].

We have employed the MMP to characterize the responses of different cell lines to various toxins and metabolic agents [17]. We report here the ability of the metabolic measurements to discriminate in real time between acute exposure of a neuroblastoma cell line to ricin toxin (RT) and botulinum neurotoxin A (BoNT/A). In addition, we report metabolic discrimination between the two pore-forming agents: alamethicin (Ala) and anthrax protective antigen (PA) in RAW macrophages, and of cholera toxin (CT) from muscarine (MU) in CHO cells. While it would be ideal to have a complete matrix of all toxins in all cell lines, we have chosen specific cell lines for this study with individual toxins in order to maximize potential metabolic changes based on the known activities of the toxins on these specific cell lines.

## 2. Results and Discussion

### 2.1 Ricin Toxin (RT)

Ricin toxin consists of two peptide chains, A_267_ and B_262_, linked by a disulfide bond. The toxin B chain reversibly binds to galactose residues of cell surface glycolipids and glycoproteins and mediates uptake of the toxin by endocytosis. The toxin A chain is an *N*-glycosidase that removes a specific adenine from 28 S ribosomal RNA [18], preventing the binding of an elongation factor, and leaving the ribosome incapable of protein synthesis [19].

**Figure 1.**
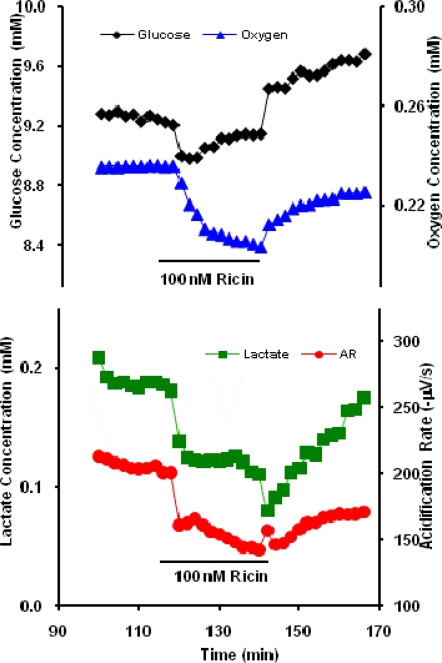
Representative neuroblastoma response in the MMP to 100 nM Ricin. Glucose and oxygen extracellular concentrations are inversely proportional to cellular uptake rates.

Neuroblastoma cells were allowed to equilibrate in the stop-flow RPMI running buffer of the MMP before exposure to 100 nM RT for 22 min. The cellular responses in the MMP show an increase in glucose uptake (GU) and oxygen uptake (OU) and a decrease in lactate production (LP) and ECAR during the time of exposure to RT (Figure 1). One explanation for this behavior may be that GU is shunted to other pathways than the production of lactate. The decrease in ECAR is likely linked to the decrease in LP, since this tends to dominate the ECAR. The cell response was rapid with all parameters showing a significant change within the first two minutes after exposure to ricin. The response is also dependent on the acute presence of ricin since oxygen, lactate, and acidification parameters show partial recovery toward baseline levels after removal of ricin from the running buffer while glucose consumption significantly decreases even from the initial baseline (Figure 1). Clearly, the lingering effects of ricin exposure may result in significant metabolic changes from the baseline, and can be explored further as part of the toxin profile in the future.

### 2.2. Botulinum Neurotoxin A (BoNT/A)

**Figure 2.**
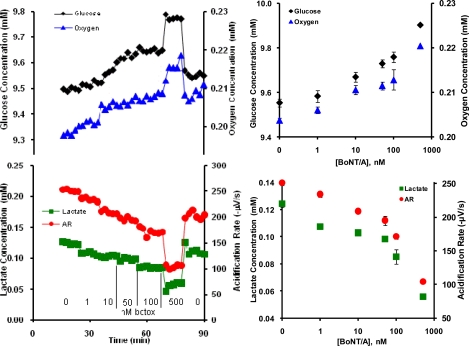
Representative BoNT/A kinetic metabolic profiles and partial dose response curves in [substrate] vs. log [BoNT/A] with sequential increasing concentration of BoNT/A.

The toxicity of BoNT/A is associated with disruption of synaptic vesicle function and prevention of neurotransmitter release [20]. Neuroblastoma cells were exposed to doses of BoNT/A (1, 10, 50, 100, and 500 nM), which was increased sequentially in the MMP cell media. We constructed cellular dose response curves (Figure 2) by averaging the MMP cell response data points (see supplemental material) related to each BoNT/A addition. Compared to the cells’ basal metabolic response, the metabolic rates for all four parameters show a step-wise decrease as a function of increasing BoNT/A dosage.

The effect of BoNT/A on undifferentiated neuroblastoma SH-SY5Y cells has been reported to inhibit the production of [^3^H]-noradrenaline ([^3^H]-NA) and cleave synaptosomal-associated proteins of 25 kDa (SNAP-25), but not cleave vesicle-associated membrane proteins [21–23]. Dose-response curves reported for the inhibition of [^3^H]-NA production and cleavage of SNAP-25 using a BoNT/A concentration from 0.3 to 1000 nM [21] correlates well with the marked decrease in GU, OU, LP and ECAR responses at concentrations of 100 nM and higher shown here. When cell exposure to BoNT/A was discontinued, the metabolic flux rates of all four analytes rapidly returned to near basal metabolic levels.

### 2.3. Alamethicin

Alamethicin is a 20 amino acid antibiotic peptide that self-assembles to form 4–8 mer ion channels [24] in lipid bilayers that can mimic nerve action potential across membranes. In addition, concentrations of alamethicin at 7 μM are reported to increase the permeability of the membrane of a lipid vesicle by fifteen-fold. In our study, rat airway (RAW) macrophage cells were perifused with low-buffered RPMI in the MMP until a steady ECAR baseline was observed. Cells were then exposed to RPMI buffer containing 15 μM alamethicin for 20 minutes, followed by RPMI with no alamethicin.

**Figure 3.**
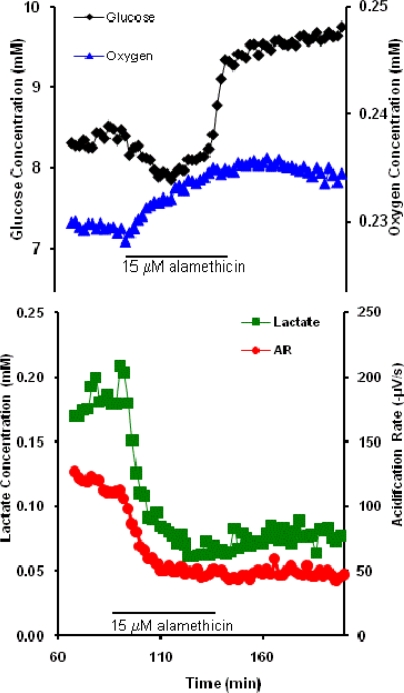
Representative effect of 15 mM alamethicin on RAW macrophage cells.

The cellular response to alamethicin on the short time scale is shown in Figure 3, and indicates an immediate decrease in the OU, LP, and ECAR. Only the GU measurement increased (seen as a decrease in glucose extracellular concentrations).

On removal of alamethicin from the perfusion medium, the cells’ response showed no recovery of OU, LP, or ECAR to previous levels, but remained essentially the same as when alamethicin was present, indicating cell necrosis (which was confirmed by staining). In contrast, the extracellular glucose consumption significantly decreased after removal of alamethicin from the running buffer to below that of the baseline rate.

A similar range of alamethicin as in this study, 25–80 μg/mL (∼12 – 40 μM, using an alamethicin m.w. = 1964.3 g/mol), led to the reduction of the membrane barrier when applied to plasma membranes of brain microsomes and erythrocyte and platelet membranes [25]. The upset of barrier function may explain the decrease in activity during perifusion with alamethicin and lack of recovery thereafter. It is unclear at this time why the glucose uptake alone would increase during exposure.

### 2.4 Anthrax Protective Antigen (PA)

*Bacillus anthracis* secretes a toxin with three components: protective antigen (PA), lethal factor (LF), and edema factor (EF). Delivery of LF and EF toxins to the cytosol is mediated by anthrax PA, which has been shown to form a heptameric prepore [10].

PA binds to a cell-surface receptor where it is cleaved into PA_20_, which dissociates into the medium, and PA_63_, which forms a heptamer, [PA_63_]_7_, that complexes with LF and/or EF. The complex is then moved through the membrane into an acidic endosomal compartment that induces a conformational change in [PA_63_]_7_, allowing insertion into the membrane to form a pore. The EF and LF are then delivered into the cytosol [26].

**Figure 4.**
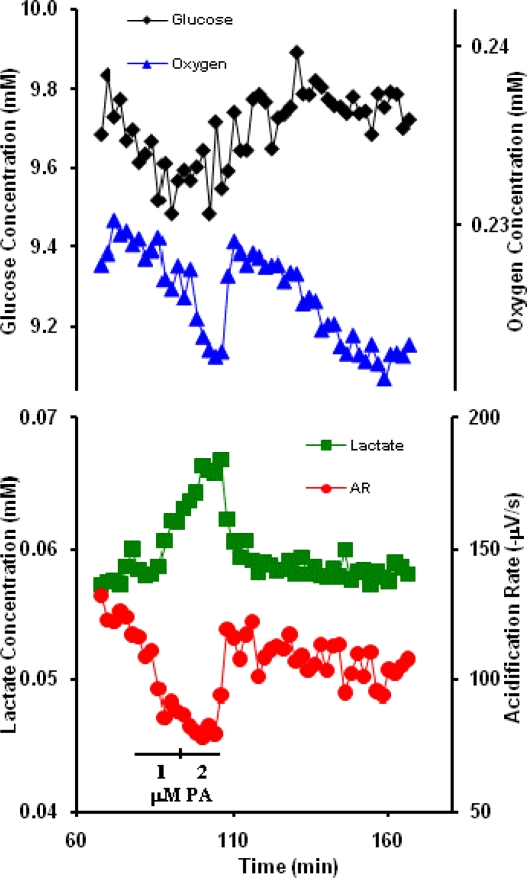
Representative RAW macrophage response to 1 and 2 μM anthrax PA.

To date there has been no report of short-term cellular effects for PA alone. We first allowed RAW macrophage cells to equilibrate in RPMI media without the toxin, until a steady ECAR baseline was observed, which was defined to be a noise of ±20 μV/s, as macrophages typically did have larger variances in acidification rates than other cell lines. The cells were then sequentially exposed for 10 minutes each to 1 and 2 μM PA, which are very high exposure concentrations. Changes in the uptake/production of the four-analytes upon addition of PA are shown in Figure 4. The concentration of extracellular glucose and oxygen can be seen to decrease during toxin addition, indicating an increase in GU and OU rates. LP shows an increase in the presence of PA while the ECAR decreases, signaling that the lactate signal can be functionally uncoupled from the total acid production signal. This uncoupling of the signal may reflect the macrophages preparing to respond to foreign biological molecules, here the PA. In this hypothesis, the LP increases as a result of increased energy usage, while the apparent ECAR decreases as a result of intracellular proton storage rather than extracellular release. This does not mean that the acidification rate of the cells decreases, only that acid is sequestered intracellularly by lysosomal storage, rather than released into the extracellular environment. In the future, dynamic metabolic pathway analysis may help with the confirmation of the identification of the exact mechanism of this uncoupling. Removal of PA from the perfusion media results in a rapid return of macrophage metabolic indicators to pre-exposure levels.

### 2.5. Cholera toxin and Muscarine

Cholera toxin (CT), produced by *Vibrio cholerae*, is an 84 kD AB_5_ hexamer consisting of five identical B subunits and a single A subunit[27]. The B subunits bind to ganglioside receptor G_M1_ and the A subunit then enters the cell. The A subunit catalyzes ADP-ribosylation of the trimeric protein G_sα_. The modified G_sα_ loses its GTPase activity, but remains constitutively active in its GTP-bound state[28], causing a continuous stimulation of adenylate cyclase. Adenylate cyclase converts adenosine monophosphate (AMP) to cyclic AMP (cAMP), which begins to accumulate in the cell[29, 30]. cAMP is necessary for the cAMP-dependent protein kinase (cAPK) activity, which targets proteins involved in glycogen metabolism. The elevated level of cAMP activates Na^+^ pumps in the lumen of the cell, forcing out Na^+^ ions. Cl^−^ ions and H_2_O then exit the cell to balance the Na^+^ release, leading to massive fluid loss, the characteristic pathology of CT.

**Figure 5.**
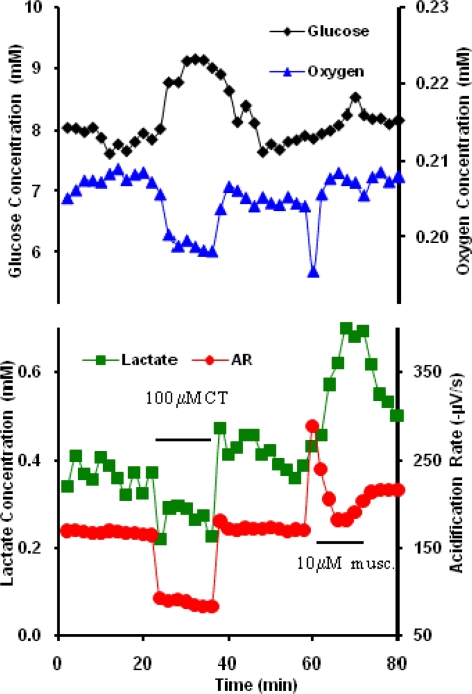
Representative CHO cell response to 1 μM cholera toxin followed by stimulation with 10 μM muscarine.

CHO cells were exposed to 1 □μM CT in RPMI for 12 minutes. On this timescale, the cholera toxin B subunit has been observed in pig enterocytes to induce the formation of numerous clathrin-coated pits and vesicles between adjacent microvilli and to appear in an endosomal subapical compartment [31]. Figure 5 shows the change in analyte concentrations during acute exposure of CHO cells to CT. OU increases while GU, LP and ECAR all show a decrease on cell exposure to CT. The observed decrease in GU is consistent with earlier observations in neuronal cells [32]. The decrease in LP and ECAR may be consistent with CT stimulating the production of adenylate cyclase, which would lead to metabolism of small glycogen stores, characteristic of CHO cells [33]. The OU rate increase may be a result of the increased cellular metabolic demand [34], such as an increase in production of cAMP, protein synthesis-dependent prostaglandin formation [35,36], and morphological elongation[36,37]. A correlation between increased OU and intestinal volume secretion was seen during exposure to CT in autoperfused segments of cat ileum [34]. In this study, the OU rate increased due to the increased metabolic work incurred from the CT induced secretory states of the small intestine, which was larger than the metabolic work of the absorptive state.

After removal of CT from the perfusion media, the cells essentially returned to pre-exposure levels, after which we stimulated them with 10 μM muscarine to check for possible antagonist behavior or activation of similar pathways of CT. Exploring toxin antagonist behavior is readily possible after exposure by simulating with a native agonist like muscarine. In response to muscarine, OU and ECAR significantly increased before rapidly returning to previous levels, while the GU and LP show a somewhat slower response (increase) followed by a return to baseline rates. Here the LP does not specifically track the immediate ECAR increase, but increases for several cycles after the ECAR spike before returning to baseline. This may be due to the production of arachidonic acid [38], which would tend to increase ECAR, yet not be detected at the lactate electrode. This ECAR spike was smaller after CT exposure than without previous exposure. Thus, in addition to direct metabolic effects by the toxin, toxins acting as antagonists can also be evaluated by multianalyte microphysiometry.

### 2.6 Discussion and Interpretation

This study was designed to determine the ability of dynamic, multianalyte microphysiometry to discriminate between toxins. Comparison of the metabolic flux responses of neuroblastoma cells exposed to RT and BoNT/A revealed a differing response in the glucose and oxygen consumption rates, with RT causing an increase in both and BoNT/A causing a decrease in all four metabolic analytes. Anthrax PA was distinguishable from alamethicin by observing changes in the macrophage oxygen and lactate metabolic fluxes despite the fact that both are considered “pore-forming” proteins. In both cases, the GU increased and ECAR decreased, but the PA caused a differential increase in OU and LP. The response of CHO cells to CT was also different than stimulation by MU, and that which we reported previously [17] for DNP and NaF. NaF exposure decreased the metabolic rates of all four analytes, while CT increased only OU. DNP (at lower concentrations) increased both LP and ECAR, while MU increased the flux rates of three of the analytes - OU, LP, and ECAR. This case shows discrimination between four agents in the metabolic responses of CHO cells.

**Figure 6.**
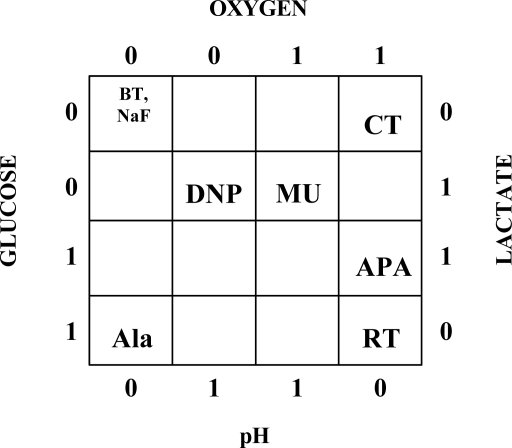
Binary four-by-four Karnaugh map of toxin responses. A ‘0’ indicates a decrease and a ‘1’ indicates an increase in the rate of consumption/production of analyte in response to the indicated analyte. BT = botulinum neurotoxin A, CT = cholera toxin, DNP = 2,4-dinitrophenol, MU = muscarine, APA = anthrax protective antigen, Ala = alamethicin, RT = ricin toxin, NaF = sodium fluoride. DNP, NaF from ref. [17].

**Figure 7.**
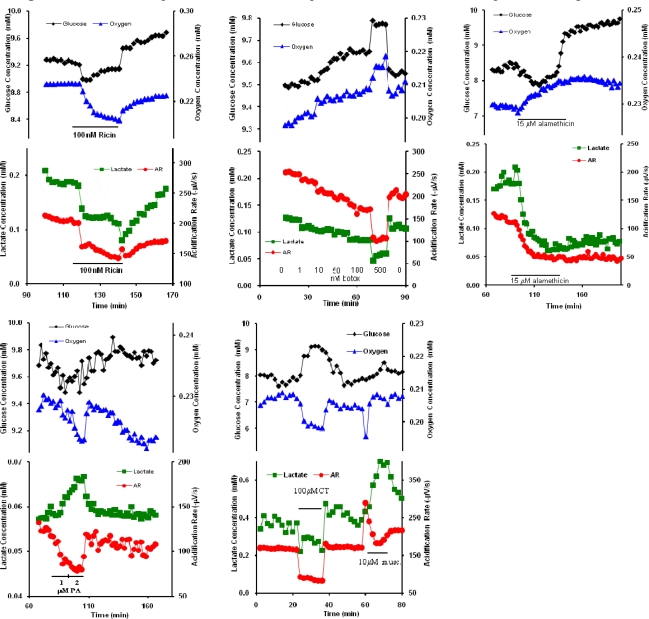
Combined representative metabolic profiles of all toxins for comparison in Figure 6.

While the absolute magnitudes of the metabolic effects observed can vary as much as 20% for each experimental replicate, the overall trends are reproducible. If we plot the different responses of the cells to each of the metabolic agents as a variable Karnaugh map (logic table), where the analytes are the four axes, we can see as a simple map (Figure 6) of metabolic response from all of the different patterns (Figure 7).

Here, a decrease in metabolic activity associated with a particular agent is assigned a ‘0’, whereas an increase is assigned a ‘1’. The map reveals a simple differentiation between each of the various agents based on the summary of the cellular response across each of the four metabolic parameters. Although we have employed different cell lines to discriminate between the agents tested here, our working hypothesis is that the selection of different cell lines will give different response profiles for all the list agents. The ideal is to have one map for each cell line at a given analyte concentration. We are presently examining the exposure of select cell lines to each agent in order to detect those responses that are unique to a particular agent, since not all cell lines are affected by agents in the same way[39]. Additional parameters, such as cell type, dose-response and temporal dynamics of the onset and recovery response could add further dimensions of discrimination.

One would expect that GU, OU, LP and ECAR reflect some degree of covariance, where changes in one parameter will necessarily alter the others due to an upset in the basal metabolism of the cells. One would predict *a priori* that GU - OU and LP - ECAR parameter pairings would reflect strong covariant relationships. Our results show that bioagents effect an unexpected dissociation between the metabolic parameters. Both Cholera toxin and alamethicin effect dissociation between GU and OU while anthrax PA functionally uncouples the expected correlation between LP and the overall ECAR. The Karnaugh map also does not take into account the temporal dynamics of agent exposure, which can add a further dimension to agent discrimination. The potential for temporal dynamics to aid agent discrimination is illustrated in the case with MU exposure, where the changes in GU and LP occur several measurement cycles behind the OU and ECAR. Dose-response relationships will also be important predictors since different concentrations can elicit different metabolic responses, such as with DNP, where low concentrations increase the LP and ECAR parameters, while high concentrations decrease all four metabolic rate measurements. Addition of these factors to a metabolic profile of agent effect on cells may create more complex ‘biosignatures’ and greatly extend the range of agent discrimination within and between cell lines [40].

With the exception of alamethicin and cholera toxin, little is known about the cellular mechanisms through which acute exposure to ricin, botulinum neurotoxin A, and protective antigen affect general cell metabolism. Interpretation of these data would be significantly enhanced by the use of an inverse dynamic metabolic model, wherein the interconnections and temporal relations between known metabolic and signaling pathways could be employed to identify agent specific mechanisms of cell toxicity. However, quantitative, dynamic analysis of the data shown in Figures 1–3 and 5 would require an analytical model that would allow for the deconvolution of lateral diffusion within the measurement chamber and sensor response kinetics. These results will be further enhanced by additional statistical analysis of the metabolic responses. Given the limitations in the access to larger quantities of select list agents, it is not possible to include a complete statistical analysis of all of the metabolic data at this time, but we believe that the general trends of metabolic responses are enough to show cell-based agent detection and at least partial discrimination of these select list agents.

## 3. Experimental Section

### Materials and Reagents

Glucose oxidase (GOx, *Aspergillus niger*), lactate oxidase (LOx, *Pediococcus*), bovine serum albumin (BSA, Fraction V, 96%), glutaraldehyde (glutaric dialdehyde, 25 wt.% solution in water), and alamethicin (from *Trichoderma viride*) were from Sigma. RPMI 1640 buffer and all Cytosensor consumable materials were from Molecular Devices Corporation (Sunnyvale, Ca). Phosphate buffered saline (PBS, 1 mM in phosphate, pH 7) was prepared fresh from stock solutions. Nafion (Perfluorosulfonic acid-PTFE copolymer, 5% w/w solution) was from Alfa Aesar, sodium fluoride (>98%) was from Fluka, and the platinum wire (24 and 36 gauge, 99.9%) was from VWR. Botulinum toxin A (from *Clostridium botulinum*), Cholera toxin (whole, A&B subunits from *Vibrio cholerae*), and Ricin communis agglutin (RCA60, includes both A&B chains) were from Sigma Chemical. Anthrax PA was provided by Jim Crowe, Vanderbilt Pediatric Immunology and Southeast Regional Center of Excellence in Biodefense (SERCEB). Neuroblastoma cells (ATCC HTB-11, Human SK-N-SH) were cultured in Eagle’s Minimal Essential Medium with Earle’s BSS and supplemented with 2 mM L-glutamine, 1.0 mM sodium pyruvate, 0.1 mM nonessential amino acids, 1.5 g/L sodium bicarbonate and 10% fetal bovine serum. The cells were cultured to confluence in 125 mL flasks under 5% CO_2_ and passaged twice before being seeded in the Cytosensor capsules. Cells were prepared for Cytosensor use by dissociating the culture with 0.25% Trypsin/0.03% EDTA, diluted to 15 mL with growth medium and centrifuged at 5000 rpm for 5 min. The cells were then re-suspended in growth medium to achieve ∼5×10^5^ cells/mL and added to the Cytosensor inserts to a final concentration of ∼2.5×10^5^ cells per insert. The inserts were incubated overnight at 30°C in a 5% CO_2_ atmosphere. RAW 264.7 macrophage cells (∼5×10^5^ per insert, ATCC TIB-71) were cultured in RPMI 1640 adjusted with 2 g/L sodium bicarbonate (Sigma), with 10% fetal bovine serum and 100 units/mL penicillin and streptavidin. CHO cell lines (ATCC # CRL-1981 M3WT4; CHO, Chinese hamster ovary) were cultured in Ham’s F12 medium with 0.05 to 0.1 mg/ml G418, 90%; fetal bovine serum, 10%. All cell lines were seeded using the same procedure above.

### Sensor Head Construction

Cytosensor Microphysiometer plunger heads were modified with platinum sensors as described previously[17,41]. Briefly, platinum wires (24 gauge) were sealed into holes drilled through the body of a typical Cytosensor^®^ Microphysiometer plunger head. The oxygen electrode was a smaller platinum wire (36 gauge) attached by silver epoxy (Epoxy Technology, Inc., Billerica MA) to the larger Pt wire. Polyurethane (Crystal Clear 200 from Smooth-On, Inc.) was used to seal the back of the electrode to provide electrical insulation and mechanical protection, with a hole left for the Cytosensor plunger. The electrodes were then polished flush with the surface of the plunger face. The microphysiometer chamber had a total volume of ∼3.5 microliters, of which approximately 1 microliter was filled by the biological cells’ volume.

### Enzyme Solutions

The oxidase enzyme solutions were prepared similarly to that described previously [17, 41]. For the oxidase film solutions, 50 mg of BSA was dissolved in 700 μL of 50 mM phosphate buffer. For the GOx solution, 5 mg of GOx was thoroughly dissolved in 600 μL of the BSA solution then quickly mixed with 6 μL of 25% glutaraldehyde solution. For the LOx film, ∼ 0.25 mg of LOx was dissolved in 70 μL of the BSA solution then quickly mixed with 0.7 μL of 25% glutaraldehyde solution. The solutions were prepared fresh when needed.

### Electrode Film Preparations

The modified sensor head electrodes were first rinsed with ethanol and distilled water. As described elsewhere[17,41], the enzyme electrode films were then prepared by allowing a droplet of the enzyme solution to dry on the platinum electrode surface. The LOx/BSA film was used as prepared in this manner. The GOx/BSA film was further modified by coating with a droplet of 5% w/w Nafion solution. A droplet of the 5% Nafion solution was also applied to the oxygen electrode (127 μm bare platinum wire) to reduce biofouling as shown in the literature [16,42,43]. The sensor heads with the modified electrodes were stored dry at room temperature when not in use, and were useful for measurements for more than 2 months.

### Electrochemical Measurements

The acidification rates were obtained with the sensor and electronics internal to the Cytosensor^®^ Microphysiometer (Molecular Devices, Inc.), while the glucose, lactate and oxygen measurements were obtained using a CHI 1030 Multipotentiostat (CH Instruments, Austin, TX). The glucose and lactate electrodes were set at +0.6 V in the ‘amperometric *i-t* curve’ to oxidize hydrogen peroxide. The oxygen electrode was set at −0.45 V to reduce dissolved oxygen or traces of other partially reduced oxygen species like hydrogen peroxide. All potentials were set versus the Cytosensor Ag/AgCl reference electrode in the effluent stream.

### Apparatus Operation

The acidification rates were obtained in the usual manner using the microphysiometer plunger and pump-on/pump-off program (80 s pump-on, 40 s pump-off). The glucose, lactate, and oxygen electrodes added to the plunger were attached to the CHI 1030 working electrodes, while the remaining platinum electrode on the sensor head was used as the CHI 1030 counter electrode. Both instruments shared the same reference electrode and experienced the same pump-on/pump-off program as applied by the microphysiometer. Minimal noise was encountered with the electrodes attached in this manner.

### Data Interpretation

The ECAR data was recorded in the usual manner from the Cytosensor, which was plotted as -μV/s vs. time (s). The changes in GU, OU, and LP rates were estimated by calculating the area under the curve during pump-off. The areas were calculated from zero current for glucose and oxygen, while the lactate area was calculated from the current baseline during flow. The normal decay current for the entire curve was corrected for by dividing the pump-off curve area by the pump-on baseline just before that respective curve. The resulting data points were then plotted as signal vs. time. The glucose and lactate signals were converted to concentrations by adding internal calibrations of glucose (1 mM aliquots) and lactate (0.1 or 0.2 mM aliquots) at the end of the experiment. The increase in signal during pump-off, as measured from the original flow baseline, was then used as the analyte calibration. The flow baseline was assumed to be the original solution concentration for each analyte with ∼10 mM for glucose, 0 mM for lactate, and ∼0.24 mM for oxygen. In this manner, the glucose and oxygen signals are inversely proportional to the cellular consumption rate. Therefore, a decrease in signal for the glucose and oxygen electrodes would be interpreted as a decrease in glucose and oxygen solution concentration, and thus an increase in the rate of cellular glucose and oxygen consumption. For the lactate electrode, a decrease in signal relates directly to a decrease in cellular lactate production.

## 4. Conclusions

Our results have shown that the multianalyte microphysiometer can be a powerful tool in the characterization of cellular metabolic activity after acute exposure to biological toxins. In addition, we have shown that toxins can produce unique profiles of cellular metabolic responses that permit discrimination between agents based on the cellular response to exposures measured in minutes versus hours. Dynamic metabolic flux analysis, coupled to the MMP data, should prove useful in building metabolic biosignatures for each selected agent. This may require the incorporation of more specific probes for analytes such as potassium, calcium, NO, glutamate, amino acids, etc. MMP technology can be readily applied to the construction and monitoring of heterogeneous cell-based analytical platforms and afford greater confidence in assessing/predicting toxicity of exogenous drugs across different cell populations. Further development of metabolic profiling using the MMP technology may eventually lead to a new cell-based sensor system for toxin identification based on the proof of concept presented here.
